# Scrub Typhus Outbreak among Soldiers in Coastal Training Area, Australia, 2022

**DOI:** 10.3201/eid3014.240056

**Published:** 2024-11

**Authors:** Rebecca Suhr, Samantha Belonogoff, Fiona McCallum, James Smith, G. Dennis Shanks

**Affiliations:** Australian Defence Force Malaria and Infectious Disease Institute, Enoggera, Queensland, Australia (R. Suhr, S. Belonogoff, F. McCallum, J. Smith, G.D. Shanks); Queensland Health, Brisbane, Queensland, Australia (S. Belonogoff, J. Smith)

**Keywords:** Scrub typhus, outbreak, soldiers, Australia, bacteria, infection, *Orientia tsutsugamushi*, Rickettsia, Leptotrombidium, chigger

## Abstract

A scrub typhus outbreak occurred among 24 soldiers from 2 Australian Defence Force infantry units following separate training events conducted in the same coastal location in tropical North Queensland, Australia, in June 2022. Seven soldiers visited a hospital, 5 requiring admission. Outbreak recognition was hampered by the geographic dispersion of soldiers after the exercise and delayed case identification resulting from such factors as prolonged incubation, cross-reactive serologic responses to other pathogens, the nonspecific symptoms of scrub typhus, and the illness’s nonnotifiable status in the state of Queensland. Our investigation focused on personal protective measures in a subanalysis of 41 soldiers, revealing an association between scrub typhus infection and the use of doxycycline chemoprophylaxis and permethrin uniform dipping.

Scrub typhus is a bacterial infection caused by *Orientia tsutsugamushi* of the *Rickettsia* family, transmitted to humans by the bite of an infected *Leptotrombidium* species chigger (larval trombiculid mite) (*1*). After an incubation period of 6–21 days, scrub typhus can cause such symptoms as fever, headache, rash, myalgia, gastrointestinal upset, lymphadenopathy, and occasionally a characteristic eschar or skin ulcer (*2*). Reports have estimated the median mortality rate of uncomplicated, treated scrub typhus to be 1.45%. However, if untreated, severe complications can develop, and mortality rates can be as high as 24% (for patients with associated multiorgan failure) and 14% (with patients with associated meningoencephalitis) (*2**–**4*). Early diagnosis is often difficult and delayed, given the nonspecific symptomatology and cross-reactive serology associated with the illness, which can be consistent with various other pathogens endemic to areas such as tropical North Queensland in Queensland, Australia (*5*).

Scrub typhus is a neglected tropical disease and a serious public health problem, and ≈1 million cases are estimated to occur annually (*2*)*.* Globally, scrub typhus is considered a rural disease endemic to dense, vegetative areas within (and sometimes beyond) (*6*) a geographic triangle that is specific to the Indo-Pacific region but can extend to include Japan, Afghanistan, and northern Australia. In Australia, accurate disease surveillance is lacking because scrub typhus is only notifiable in Western Australia. The disease is likely underdiagnosed in the North Queensland regional areas (*7*), where case reporting is predominantly limited to military outbreaks at Cowley Beach Training Area (CBTA) (*8**–**10*) (Figure 1).

**Figure 1:**
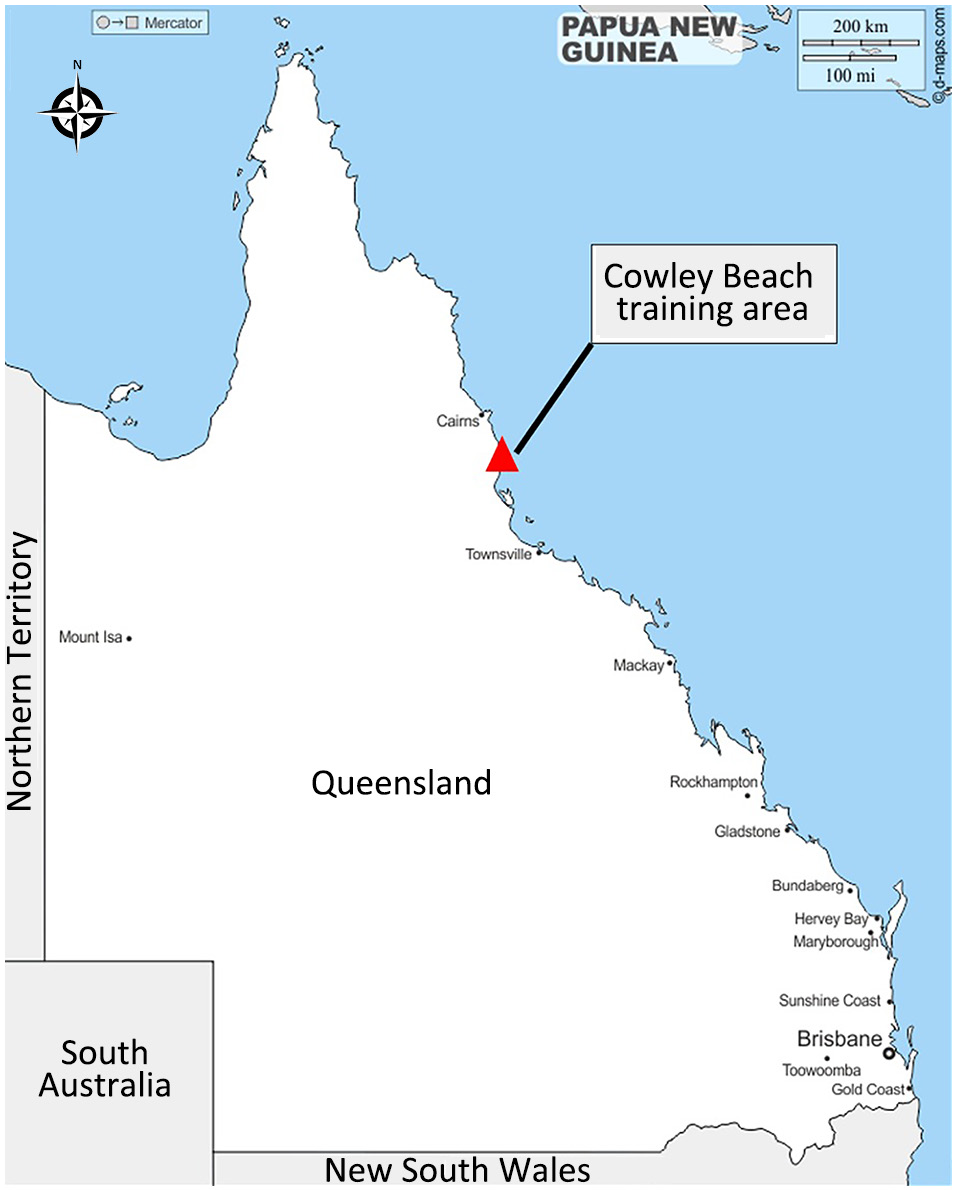
Location of Cowley Beach Training area within the state of Queensland, Australia.

Scrub typhus emerged as a significant disease for Australian and allied militaries deployed to southeast Asia and southwest Pacific regions during World War II (*11*), and, in the absence of antibiotic treatment options, scrub typhus infections greatly affected military capabilities, resulting in attrition as high as 9% (*12*)*.* Despite the development and introduction of chloramphenicol and chemical treatment for uniforms after the war, scrub typhus remains a serious vectorborne disease that continues to threaten soldiers training in jungle and coastal environments (*13**–**15*). Our report of a recent outbreak of this illness highlights this threat.

In July 2022, the Australian Defence Force Malaria and Infectious Disease Institute assisted in the investigation of an emerging cluster of febrile soldiers in 2 infantry units from Brisbane and Townsville, Queensland, Australia (designated Brisbane-based and Townsville-based), that had recently returned from different exercises at the CBTA. Three soldiers received diagnoses of laboratory-confirmed scrub typhus in the early phase of the outbreak, but positive serologic results prompted physicians to consider other diagnoses as well, including leptospirosis, Q fever, and Japanese encephalitis virus. Our research explores the challenges of confirming a scrub typhus outbreak and provides statistical analysis of doxycycline chemoprophylaxis and permethrin treatment of clothing in preventing scrub typhus infection.

## Methods

During the 2022 scrub typhus outbreak, the Australian Defence Force Malaria and Infectious Disease Institute staff communicated with treating military-based clinicians, Public Health Units, and diagnostic laboratories regarding the number of symptomatic persons, diagnostic tests being performed, and those test results. Existing military- and civilian-derived medical records and pathology results for affected soldiers were sourced and reviewed, with further testing requested if required.

Scrub typhus diagnosis is confirmed via positive nucleic acid testing of blood or eschar biopsy, seroconversion, or a 4-fold rise in total antibody titers in serologic testing (*2*). Confirmed and probable cases of scrub typhus in this outbreak were determined by using the Western Australia case definition (*16*) (Appendix). A clinical case definition was also established to capture soldiers with a compatible illness and epidemiologic links to the outbreak but with no disease cause identified through laboratory testing. We determined exposed, noncase soldiers, defined as infantry soldiers who conducted the same activities as case patients in the same environment but did not become ill, by using unit nominal rolls of personnel involved in the exercises.

Our research aimed to compare disease-risk data for a subset of personnel with scrub typhus (Brisbane-based case-patients) and a control group of exposed, noncase soldiers. Researchers interviewed scrub typhus case patients and asked exposed, noncase soldiers to complete an anonymized questionnaire. Questions included details regarding training area movement and activities, level of compliance with individual protection measures, and barriers to prophylaxis compliance. We entered data into an electronic database (Microsoft Excel 2016; Microsoft, https://www.microsoft.com) on a restricted military network and used Stata version 14.0 statistical software (StataCorp LLC, https://www.stata.com) for analysis. We used a χ^2^ test of independence to investigate use of uniform permethrin dipping and doxycycline prophylaxis for case-patients versus noncase personnel. Because of a very low response from Townsville-based, noncase personnel, we did not include the 12 Townsville-based case-patients in our statistical comparison of data.

## Results

We identified 24 cases of scrub typhus among military members from the 2 infantry units after training exercises conducted at CBTA during June 7–24, 2022. We determined a total of 337 soldiers from both units to be exposed personnel. Scrub typhus attack rate was 18.8% (12/64; 5 confirmed cases, 6 probable cases, 1 clinical case) for the Brisbane-based cohort conducting jungle warfare training and 4.4% (12/273; 8 confirmed cases, 3 probable cases, 1 clinical case) for the Townsville-based cohort conducting amphibious landings.

**Figure 2:**
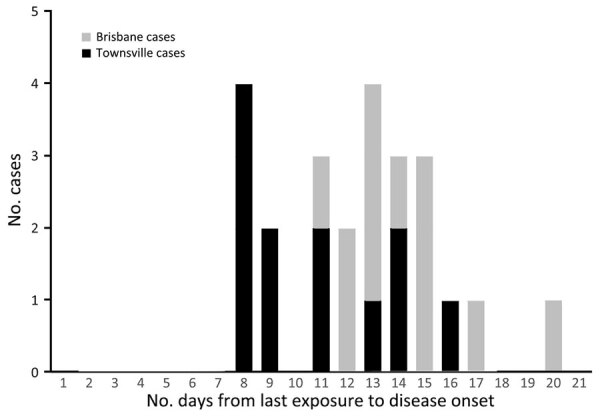
Epidemiologic curve of the 2022 scrub typhus outbreak occurring at Cowley Beach Training Area, Queensland, Australia. Training participants were from either Brisbane or Townsville in Queensland.

The incubation period range for the 24 cases, determined from the last possible date of exposure at CBTA to the onset of illness, was 8–20 days (median 12 days) (Figure 2). None of the soldiers had received a prior diagnosis of scrub typhus. Seven soldiers (29%, 7/24) visited a hospital, 5 (21%, 5/24) of whom were admitted to the hospital for an average length of stay of 5.6 days (range 2–8 days). Symptoms included seizures caused by meningoencephalitis (requiring intensive care unit admission), multisystem inflammatory response with evidence of hemodynamic instability, pulmonary congestion (requiring high-flow oxygen supplementation), acute kidney injury and hepatosplenomegaly, and uncomplicated cases of electrolyte disturbances and jaundice. The mean age for the soldiers was 27 years, and all were physically fit, with few or no comorbidities. Of the hospitalized soldiers, we categorized 4 as having a confirmed case of scrub typhus and 3 as having a probable case of the illness (maximum *O. tsutsugamushi* titer demonstrated on initial blood testing and therefore no capacity to demonstrate seroconversion or a rise in titer). We compiled data relating to symptom onset, blood sampling, and *O. tsutsugamushi* serologic results for all 24 case-patients (Appendix Table 2). We noted headache and fever to be the most common symptoms reported (Table), a finding consistent with previous literature (*2*). All soldiers responded well to treatment with doxycycline, and no soldiers died as a result of this outbreak.

**Table:** Common symptomatology among 24 case-patients investigated during a scrub typhus outbreak at Cowley Beach Training Area, Australia, 2022.

Symptom or sign	No. (%) case-patients
Headache	20 (96)
Fever	22 (92)
Abnormal liver function*	18 (86)
Rash	16 (67)
Myalgia or arthralgia	18 (75)
Fatigue	14 (58)
Eschar or bites	13 (54)
Nausea or vomiting	12 (50)

*Three case patients did not have liver function checked

At the time of illness onset, some soldiers were on leave and dislocated from their home unit location, delaying initial assessment. In addition, several soldiers were dispersed once they clinically improved, creating challenges in obtaining the blood samples required for serologic investigation. We conducted scrub typhus nucleic acid testing by using primary blood samples from 17 (71%) of the 24 soldiers. We collected 14 samples beyond the recommended 5-day window from illness onset; however, 3 (18%, 3/17) of the 14 were positive; of note, those samples were from hospital testing.

**Figure 3:**
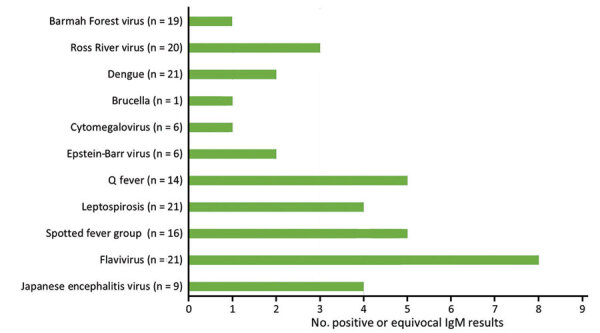
Cross-reactive positive or equivocal IgM serologic results in cohort of 24 scrub typhus cases investigated during a 2022 outbreak at Cowley Beach Training Area, Queensland, Australia. n values indicate number of case-patients tested for each pathogen/disease.

Many soldiers had positive IgM serologic responses to other pathogens. We observed 36 positive or equivocal IgM results for 11 diseases (Figure 3) for 17 of the 22 case-patients who underwent serologic testing. Results for the case patients who were retested revealed no increase in titer or immunological progression to an IgG response to other pathogens. Because a scrub typhus outbreak was eventually confirmed, we assumed that all of those results were related to IgM cross-reactivity between pathogens. Clinical improvement of all cases after commencement of treatment with doxycycline supported a diagnosis of scrub typhus, although that treatment is also appropriate for some differential diagnoses, such as leptospirosis and Q fever (*17*).

**Figure 4:**
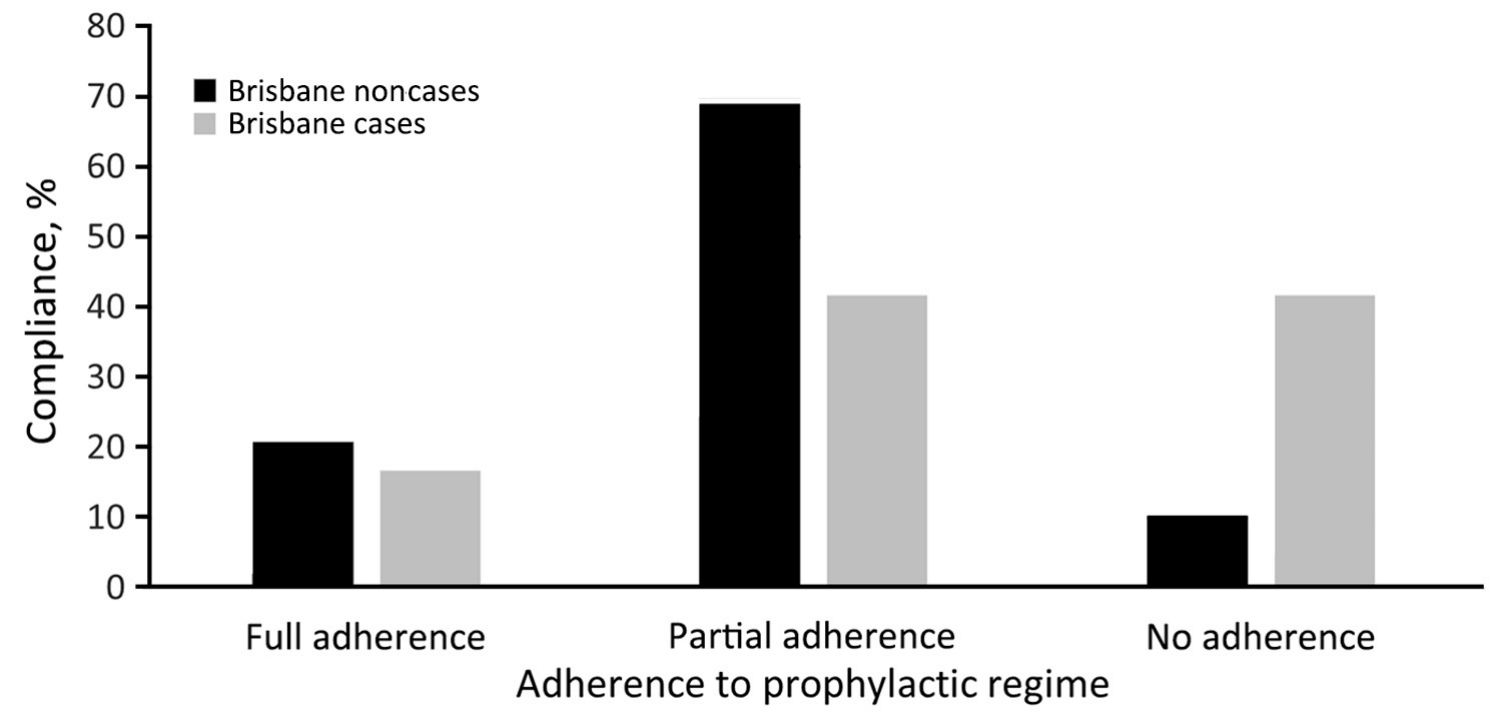
Reported adherence to prophylactic doxycycline among 12 case patients from a Brisbane, Australia–based military unit compared with 29 Brisbane-based exposed, noncase soliders obtained as part of an investigation of scrub typhus at Cowley Beach Training Area, Queensland, Australia, 2022.

All personnel who attended the exercises at CBTA received prophylactic doxycycline. We gathered self-reported adherence data for 64% (41/64) of the Brisbane-based soldiers who participated in jungle training and for 56% (29/52) of the exposed, noncase soldiers (Figure 4). Most soldiers reported the intention to comply with the regimen. Circumstances contributing to omitted doses included irregular or absent food intake, damage to tablets (i.e., wet or crushed) in packs, secondary health complications (e.g., gastric reflux, vomiting), and loss of routine. Seven (58%) of 12 Brisbane-based case patients and 26 (90%) of 29 exposed, noncase soldiers reported complete or partial compliance (>1 tablet taken while at CBTA) with the doxycycline regimen. Analysis showed that any doxycycline intake was protective and that soldiers who did not take any doxycycline were 6 times more likely to be infected with scrub typhus than those who took any doxycycline (OR 6.2, 95% CI 1.2–33.3; p = 0.03).

Case-patients and Brisbane-based exposed, noncase soldiers generally adhered to supplied personal protective measures. Questions regarding the appropriate wearing of uniforms and use of DEET insect repellent garnered consistent (>90%) “well adhered to” responses from both case-patients and noncase personnel. Of note, all scrub typhus case-patients reported sleeping or lying on the ground, compared with 72% of the Brisbane-based exposed, noncase soldiers. We collected information regarding uniform dipping for 36 Brisbane-based soldiers (9 case-patients; 27 exposed, noncase soldiers), revealing an overall compliance of 83.3% (30/36), including 5 of the 9 case-patients having dipped their uniforms. Data suggested that uniform dipping was protective, and soldiers who did not wear dipped uniforms were 10 times more likely to be infected with scrub typhus than those who wore dipped uniforms (OR 10, 95% CI 1.42–71.43; p = 0.021). Soldiers reported varying tolerability and attitudes toward doxycycline prophylaxis compliance and barriers to individual protection measure adherance.

## Discussion

This most recent outbreak of scrub typhus at an Australian military training area underscores the continuing propensity of this illness to hinder military operations throughout the Indo-Pacific region. Despite established prophylactic regimens among military personnel at CBTA, the 2 infantry units involved in this outbreak had scrub typhus attack rates of 18.8% (Brisbane-based cohort) and 4.4% (Townsville-based cohort). The higher attack rate in the Brisbane-based cohort might reflect more intimate exposure to vegetation during jungle warfare training, and hence to mites, when compared with activities associated with amphibious landings.

Several factors contributed to a delay in the identification of the outbreak. Disease manifestations in all cases commenced >1 week after exercise completion, when a large proportion of personnel were on leave, many having traveled interstate. The scattered nature of personnel and unavailability of blood samples and medical and pathology records contributed to the delay in paired serology for some patients. In addition, some soldiers were unwilling or unable to undergo further laboratory investigation after clinical improvement.

Because testing of many ill soldiers revealed IgM seropositivity to several pathogens, serologic results of which arrived prior to those for scrub typhus, we were delayed in establishing diagnostic confirmation of the outbreak. Most clinicians requested rickettsial serology at the time of initial patient evaluation. However, the rickettsial screening offered by some pathology providers did not include *O. tsutsugamushi* serology, further delaying confirmatory results. Laboratory standard of intermittent, batched serologic testing for *O. tsutsugamushi* indirect fluorescent antibodies added to the delay. It therefore took an average of 23 days after sample collection to receive scrub typhus serologic test results for all patients.

We acknowledge several limitations of our investigation, including a relatively small case population; statistical analyses should be therefore interpreted with caution. It is possible that some nonconfirmed cases experienced a disease other than scrub typhus (e.g., leptospirosis), posing a misclassification risk to our analyses. However, when we removed the single clinical case from our analysis, the findings remained consistent and statistically significant. In addition, although we collected data regarding prophylactic compliance, we could not confirm that information, which might have influenced results.

In conclusion, this outbreak investigation confirmed a statistically significant association between scrub typhus disease occurrence and noncompliance with the prescribed doxycycline regimen, supporting the use of doxycycline chemoprophylaxis against scrub typhus disease (*18**,**19*)*.* We also noted a strong association between nonuse of permethrin-treated uniforms and scrub typhus disease occurance, consistent with existing knowledge of this preventative measure in reducing exposure to disease vectors (*20*)*.*

This outbreak highlights the need for increased awareness of this pathogen threat in both military and civilian health settings in Australia and among Pacific and Asian partners. The illness severity, even among young healthy soldiers, and occasional lethality of scrub typhus, together with reports of the illness throughout northern Australia, Southeast Asia, the Western Pacific, and China’s South China Sea regions, make scrub typhus a dangerous, lingering issue for military health planners throughout the Indo-Pacific region.

Appendix Additional information for scrub typhus outbreak among soldiers in coastal training area, Australia, 2022.
