# Supplementary material for: Scrub Typhus Outbreak among Soldiers in Coastal Training Area, Australia, 2022
Source: Emerg Infect Dis. 2024 Nov;30(Suppl 2):S41–6. doi: 10.3201/eid3014.240056 (PMC11559576; doi:10.3201/eid3014.240056)
Supplement: Appendix — Additional information for scrub typhus outbreak among soldiers in coastal training area, Australia, 2022. [file 24-0056-Techapp-s1.pdf]

Article DOI: https://doi.org/10.3201/eid3014.240056

EID cannot ensure accessibility for supplementary materials supplied by authors. Readers who have difficulty accessing supplementary content should contact the authors for assistance.

Scrub Typhus Outbreak among Soldiers in Coastal Training Area, Australia, 2022

Appendix

Western Australian Government Department of Health definition of confirmed and probable scrub typhus infection for reporting purposes:

84. Typhus or rickettsial infection (Not nationally notifiable) (includes murine typhus, louse borne typhus, scrub typhus, Queensland tick typhus, African tick typhus and the “spotted fevers”) (last updated: 2013)

Reporting

Both confirmed cases AND probable cases should be notified.

Confirmed Case

A confirmed case requires laboratory definitive evidence.

Laboratory definitive evidence: 1. Detection (culture or nucleic acid testing) of Rickettsia species or Orientia tsutsugamushi in a clinical specimen OR 2. Seroconversion or a 4-fold or greater rise in serum antibody titer to a Rickettsial or Orientia sp. group between acute and convalescent phase sera.

Probable Case

A probable case requires laboratory suggestive evidence AND clinical evidence AND epidemiologic evidence.

Laboratory suggestive evidence: A single elevated antibody titer to a Rickettsial or Orientia species group.

Clinical evidence: A clinically compatible illness (fever and at least one of headache, myalgia, rash or eschar).

Epidemiologic evidence: In the month before onset of illness, history of travel to a region (in Australia or overseas) where the detected Orientia or Rickettsia species or group is known to occur (Appendix Table 1).

Note: Some laboratories report results at the species level (eg, *Rickettsia conorii*), however, if the species is not known to occur in the place where the infection was most likely acquired (see Appendix Table 1), then it should be reported at the 'group' level (eg, a reported *R. conorii* infection that was likely to have been acquired in WA, where it has not previously been detected, would be recorded on WANIDD as "spotted fever group."

Source: Surveillance Case Definitions for Notifiable Infectious Diseases and Related Conditions in Western Australia, Communicable Disease Control Directorate, December 2023, pp 112–113.

Appendix Table 1: Classification and geographic distribution of *Orientia* species

Group	Species	Vector	Disease	Geographic distribution
Scrub typhus (1 species only)	*Orientia tsutsugamushi*	Mites (*Leptotrombidium* spp)	Scrub typhus	Northern WA, NT and QLD, overseas

WA, Western Austrailia; NT, Northern Territory; QLD, Queensland.

Appendix Table 2. Dates and results of nucleic acid testing testing and *O. tsutsugamushi* IFA titers performed on the 24 outbreak cases. Dates are described symptom onset post last possible point of exposure and for the blood draws as days from symptom onset.

			NAT		Initial collection		Secondary collection	
Case number	Symptom onset (days from exposure)	Hospital admission	Result	Days from symptom onset	*O. tsutsugamushi* IFA titer	Days from symptom onset	*O. tsutsugamushi* IFA titer	Days from symptom onset
1	+13	No	Negative	+6	256	+6	256	+26
2	+10	No	Negative	+8	ND	+8	256	+29
3	+14	No	ND	ND	128	+4	>1024	+57
4	+7	No	Negative	+5	>1024	+10	512	+82
5	+13	No	Negative	+10	<128	+4	512	+57
6	+12	No	Negative	+4	<128	+4	>1024	+65
7	+7	Yes	ND	ND	Hospital		>1024	+27
8	+7	No	Negative	+11	<128	+11	ND	ND
9	+8	No	Negative	+7	512	+7	256	+199
10	+7	Yes	Positive	+11	128	+8	ND	ND
11	+10	No	ND	ND	>1024	+7	ND	ND
12	+8	Yes	ND	ND	>1024	+8	>1024	+38
13	+13	Yes	ND	ND	>1024	+22	>1024	+33
14	+12	No	Negative	+27	<128	+5	256	+27
15	+13	No	Negative	+6	<128	+4	<128	+26
16	+15	No	ND	ND	>1024	+3	ND	ND
17	+13	No	ND	ND	<128	+5	>1024	+25
18	+11	No	Positive	+7	<128	+7	>1024	+19
19	+14	No	Negative	+4	<128	+4	512	+15
20	+12	Yes	Positive	+13	ND	ND	512	+27
21	+15	No	Negative	+7	<128	+7	512	+50
22	+17	No	Negative	+7	256	+7	256	+49
23	+15	No	Negative	+25	256	+11	>1024	+25
24	+20	No	Negative	+18	<128	+8	512	+18
